# Patients with prolonged stay on ICUs and the risk of mortality within 1-year of cardiac surgery

**DOI:** 10.1186/cc13243

**Published:** 2014-03-17

**Authors:** A Grayson, N Coulson, N Scawn

**Affiliations:** 1 The Liverpool Heart and Chest Hospital, Liverpool, UK

## Introduction

The aim of this study was to determine an appropriate risk model to identify patients at high risk of prolonged ICU stay and to aid patient consent prior to cardiac surgery.

## Methods

Data were prospectively collected on 5,440 consecutive cardiac surgery cases between April 2009 and March 2012. The primary outcome measure was the combined outcome of prolonged ICU stay (length of stay greater than 20 days) and/or in-hospital mortality. Logistic regression was performed to assess the predictability of logistic EuroSCORE against the primary outcome. Low-risk, medium- risk and high-risk groups were identified and subsequent risk of 1-year mortality assessed. Survival status was determined at 1 year.

## Results

**Figure 1:**
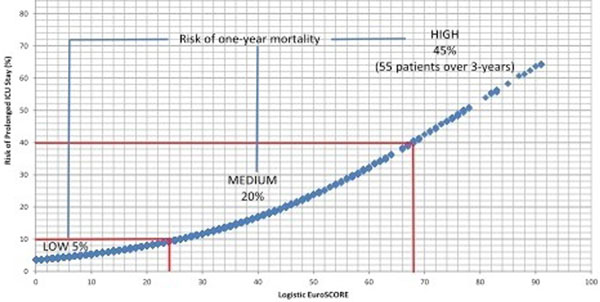
**Risk of long stay versus 1-year mortality**.

A total of 192 (3.5%) patients had a prolonged ICU stay and 187 (3.4%) in-hospital deaths occurred, resulting in a combined primary outcome of 349 (6.4%). At 1 year, 371 (6.8%) deaths occurred. The risk of death in-hospital and at 1 year was significantly higher in patients with prolonged ICU stay (in-hospital mortality, 15.6% vs. 3.0%; *P *< 0.001/1 year, 27.6% vs. 6.1%; *P *< 0.001). The mean logistic EuroSCORE for all patients was 10.9. Patients with prolonged ICU stay had a significantly higher logistic EuroSCORE (20.3 vs. 10.6; *P *< 0.001). The logistic EuroSCORE was a reasonable predictor of prolonged ICU/ in-hospital mortality (OR 1.04, 95% CI 1.04 to 1.05, *P *< 0.001) with a receiver operating characteristic (ROC) curve of 0.72. The relationship between a patient's logistic EuroSCORE and predicted risk of prolonged ICU is shown in the figure; including low-risk, medium-risk and high risk groups. Around 50% of the entire cohort of patients had a logistic EuroSCORE of 10 or less and an associated risk of prolonged ICU stay of 5% or less. See Figure 1.

## Conclusion

Using an existing risk prediction model, a patient's risk of prolonged ICU stay can be calculated using contemporaneous data. This information could be relevant for aiding in providing informed consent for cardiac surgery patients.

